# *M*Fe_6_ *X*_4_ system (*M* = Mg, Sc, Zr; *X* = Al, Si, P, Ga, Ge, In, Sn, Sb) as possible ‘gap’ magnets

**DOI:** 10.1080/14686996.2025.2527024

**Published:** 2025-07-07

**Authors:** Alena Vishina, Rebecca Clulow, Daniel Hedlund, Vitalii Shtender, Peter Svedlindh, Martin Sahlberg, Olle Eriksson, Heike C. Herper

**Affiliations:** a Department of Physics and Astronomy, Uppsala University, Uppsala, Sweden; b Department of Chemistry - Ångström, Uppsala University, Uppsala, Sweden; c Department of Materials Science and Engineering, Uppsala University, Uppsala, Sweden; d Sweden and Wallenberg Initiative Materials Science, WISE, Uppsala University, Uppsala, Sweden

**Keywords:** Permanent magnets, magnetic anisotropy, DFT, magnetism, rare-earth-free

## Abstract

LiFe_6_Ge_4_, with a theoretically predicted saturation magnetization of 1 T, a magnetocrystalline anisotropy energy of 1.78 MJ/m^3^ and a Curie temperature of 620 K was suggested to be a promising permanent magnet as an outcome of a data-mining search. Magnetic measurements of the synthesized sample are reported here. Unfortunately, experiments revealed a weak ferromagnetic behaviour with magnetization values much below that predicted by theory. This discrepancy is analyzed in detail, and is attributed to the trigonal crystal symmetry that was missed in the previous characterisation of the material. The correct crystal structure is *R*$\bar{3}$ *mH* (space group 166) and it is found here to have an antiferromagnetic ground state, as opposed to a theoretically predicted ferromagnetic state of the previously reported monoclinic crystal structure. Theoretical calculations show that element substitution can stabilize a ferromagnetic state of the trigonal crystal structure, with high values of saturation magnetization and magnetocrystalline anisotropy. The best results are seen for the Al or Ga substitution for Ge of the LiFe_6_ *X*_4_ compound.

## IMPACT STATEMENT

Combining data-driven modeling with synthesis, we resolve the structural origin of magnetism in LiFe6Ge4 and propose element substitution strategies for sustainable, rare-earth-free permanent magnets with enhanced performance.

## GRAPHICAL ABSTRACT

**Figure:**
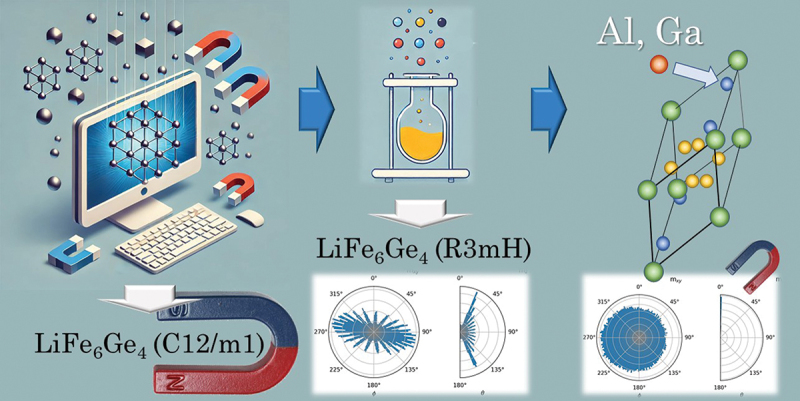

## 1. Introduction

With the rapid growth in the number of electric vehicles and windmills required for the transition to an electrified society and green energy solutions, there is an ever-increasing demand for high-performance permanent magnets (PM). All the materials used in these applications contain rare-earth (RE) elements, which are considered critical [1,2]. Various materials have been investigated in an attempt to decrease the amount [3,4] of REs or find RE-free alternatives while maintaining the necessary performance.

There is also a large gap in the price-performance space between the hard ferrite magnets with their low price and low magnetization and the expensive high-performance RE-permanent magnets [5], providing further incentive for new RE-free magnetic materials with intermediate-performance (‘gap’ magnets).

When searching for materials that possess the correct magnetic properties, one should look for ferromagnetic (FM) systems with high saturation magnetization ($M_{S}$), uniaxial magnetic anisotropy with large magnetocrystalline anisotropy energy (MAE), and high Curie temperature ($T_{C}$).

The search for new RE-free permanent magnets is currently carried out at many laboratories, both on experimental and theoretical fronts. Among the promising materials are $\alpha {\;}^{\prime }{\;}^{\prime }$-Fe${\;}_{16}$N${\;}_{2}$ and L1${\;}_{0}$-FeNi type materials [6,7], Mn-based intermetallics, such as MnBi, MnAl, MnAlGe and MnGa [8–10], Co-rich transition-metal alloys, and many more [11]. Various computational and experimental methods have been tested, such as high-throughput experiments [12], high-throughput computational screening [13], additive manufacturing [14], etc.

The current investigation is an outcome of a high-throughput search, as described in Ref. [15]. The details of the search criteria and methods were given in Refs. [16,17]. In our data-mining approach, we filter through materials of several databases, both previously synthesized (Inorganic Crystal Structure Database (ICSD) [18]) and predicted theoretically [19]. For each entry, we calculate the required magnetic characteristics ($M_{S}$, MAE, $T_{C}$, etc). LiFe${\;}_{6}$Ge${\;}_{4}$ was found in the ICSD database among the materials containing a 3 *d*- and a *p*-element of the Periodic Table [15]. It had the highest magnetization, magnetocrystalline anisotropy, and $T_{C}$ of all the promising magnetic materials discovered in the investigation.

Following the theoretical predictions, LiFe${\;}_{6}$Ge${\;}_{4}$ (and ZrFe${\;}_{6}$Ge${\;}_{4}$) was here synthesized experimentally to, firstly, attempt to reproduce the structure reported in the ICSD and, secondly, to verify the predicted magnetic properties. The experimental methods and results are described below in detail. A discrepancy between experimental results and theoretical predictions was observed and explained, as a different (to the ICSD entry) crystal structure was obtained in the experiment. The relative stability and properties of both structures were analyzed from theoretical and experimental points of view. In addition, substitutional alloying was further investigated theoretically to improve the properties of the more stable crystal form.

## 2. Computational methods

For the high-throughput search, the full-potential linear muffin-tin orbital method (FP-LMTO) as implemented in the RSPt code [20,21] was used. The PBE functional [22] for exchange and correlation was employed. The results were obtained with the tetrahedron method with Blöchl correction for the Brillouin zone integration [23,24]. Further details can be found in Ref. [15].

Structure relaxations for element substitutions were performed using the Vienna Ab Initio Simulation Package (VASP) [25,26] within the Projector Augmented Wave (PAW) method [27]. The formation enthalpy of a material was calculated as the energy difference between the material and the composition-weighted average of its elemental components. For example:

$$\Delta H[L\mathrm{iF}e_{6}Ge_{4}]=E_{LiFe_{6}Ge_{4}}-E_{Li}-6E_{Fe}-4E_{Ge}.$$

Here $E_{Li}$, $E_{Fe}$, and $E_{Ge}$ are the energies per atom of alpha-samarium structured Li, body-centered cubic (bcc) iron, and diamond structured Ge, respectively. The results are given per atom for the elements and per formula unit for the compound.

The magnetic anisotropy energy was obtained (using the RSPt code) as the energy difference $\Delta E=E^{\mathrm{pl}}-E^{c}$. $E^{c}$ and $E^{\mathrm{pl}}$ are the total energies calculated with the magnetization directed along and perpendicular to the crystallographic c-axis. A positive sign of the MAE corresponds to the required uniaxial anisotropy. A converged with respect to the number of k-points Monkhorst-Pack mesh [28] of 20×20×20 was used.

The Curie temperature $T_{C}$ was calculated employing Metropolis Monte Carlo (MC) and Atomistic Spin Dynamics (ASD) simulations as implemented within the Uppsala Atomistic Spin Dynamics (UppASD) software [29]. The simulations were performed on a 20×20×20 supercell with periodic boundary conditions. The exchange parameters $J_{\mathrm{ij}}$ were calculated with the RSPt code within the first seven coordination shells [30–32].

For the case of LiFe${\;}_{6}$Ge${\;}_{5}$, $J_{\mathrm{ij}}$s were calculated for the experimental structure with the spin-polarized Korringa-Kohn-Rostoker method [33,34] within the atomic sphere approximation (ASA) as implemented in the SPR-KKR code [35]. $J_{\mathrm{ij}}$s were calculated within a cluster of atoms with the radius of 2.5 *a*.

In addition, the Sumo package [36] and VESTA code [37] were utilized for visualisation.

## 3. Experimental methods

The sample was synthesised by heating Fe${\;}_{6}$Ge${\;}_{4}$ powder with a 200 at% excess of Lithium pieces (CEL, 99.8 %) in a tantalum tube at 1000 ${\;}^{\circ }$C for 1 hour followed by 800 ${\;}^{\circ }$C for 2 days. Fe${\;}_{6}$Ge${\;}_{4}$ powder was initially prepared from alloying of Fe (Goodfellow, purity 99 %) and Ge (Lesker, purity 99.999 %) using an arc furnace. The sample was remelted three times to ensure good homogeneity. Further preparation was conducted in a glove box and sealed under inert conditions. The Ta tube was then sealed within a steel tube to protect the vessel from oxidation at high temperature. The ZrFe${\;}_{6}$Ge${\;}_{4}$ sample was prepared by arc melting stoichiometric quantities of Zr (Goodfellow, Purity 99.2 %), Fe and Ge under an argon atmosphere. The sample was remelted three times to ensure good homogeneity. The sample was wrapped in tantalum foil before annealing at 1000 ${\;}^{\circ }$C in an evacuated silica tube for seven days. The phase purity and crystal structure of the compounds were analysed using powder X-ray diffraction using a Bruker D8 diffractometer (Bruker, USA) equipped with a lynx-eye position-sensitive detector (PSD) and Cukα radiation (λ = 1.54 Å). The data were analysed using the Rietveld refinement method within the Topas 6 software program [38].

Magnetic measurements were made using a Quantum Design MPMS XL system (Quantum Design, USA) as well as a LakeShore vibrating sample magnetometer (Lake Shore Cryotronics, USA).

## 4. Results

### 4.1. LiFe_6_Ge_4_ as the result of the high-throughput search

**Figure B1.**
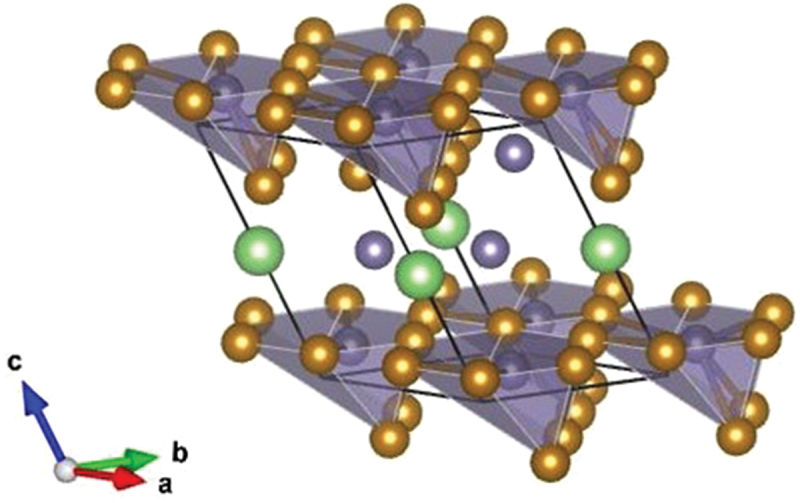
(Color online) the unit cell of LiFe_6_Ge_4_, space group 12. Iron atoms are shown with brown spheres, Ge atoms are small violet spheres, and Li is denoted with large green spheres.

A high-throughput and data-mining search for rare-earth-free permanent magnets was previously performed among the materials containing a 3 *d* and *p*-element of the Periodic Table using the approach suggested in Ref. [16]. The details are given in Ref. [15]. One of the promising materials found was LiFe${\;}_{6}$Ge${\;}_{4}$. Its calculated saturation magnetization, magnetic anisotropy energy and Curie temperature are 1 T, 1.78 MJ/m${\;}^{3}$, and 620 K, respectively [16]. The ground state was found to be ferromagnetic. The monoclinic unit cell of LiFe${\;}_{6}$Ge${\;}_{4}$ (ICSD 100,061) with *C*12*/m*1 (12) space group that was used in the theoretical calculations is shown in Figure B1.

Following the high-throughput investigation, LiFe${\;}_{6}$Ge${\;}_{4}$ was synthesized and its magnetic properties were investigated, to corroborate the theoretical findings. As reported below, the magnetization was, unexpectedly, found to be much lower than what the theory suggests [16]. The origin of this discrepancy was then explored and is found to be caused by structural effects. Moreover, following the theoretical analysis, the structure reported in the ICSD does not seem to be valid. Two approaches were further pursued to enhance the magnetism: i) a theoretical element substitution on the Li and Ge sites of LiFe${\;}_{6}$Ge${\;}_{4}$ in order to improve the magnetic state; ii) the experimental substitution of Li for Zr and Ge for Ga. Details of these results are also given below.

### 4.2. Experiment: synthesis, structure, and magnetism

**Figure B2.**
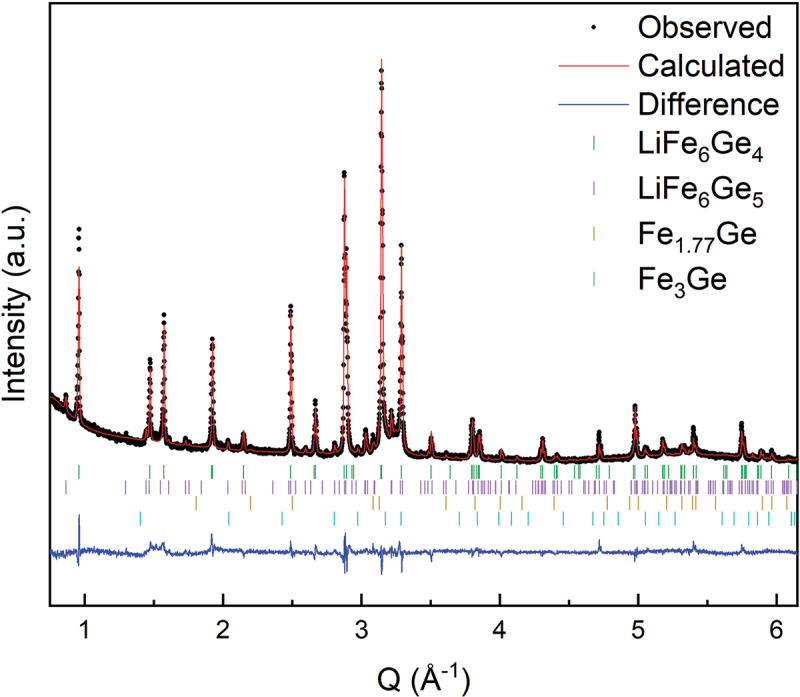
(Color online) Powder X-ray diffraction pattern (*λ* = 1.54 Å) and Rietveld refinement of LiFe_6_Ge_4_ and LiFe_6_Ge_5_. *rwp* 7.48 and *χ*^2^ 3.34. The measured diffraction data is shown in black,the calculated diffraction curve based on the structural model in red and the difference between the calculated and measured data in blue. The tick marks indicate the expected peak positions for LiFe_6_Ge_4_, LiFe_6_Ge_5_, Fe_1.77_Ge and Fe_3_Ge compounds.

LiFe${\;}_{6}$Ge${\;}_{4}$ was formed as the majority phase with LiFe${\;}_{6}$Ge${\;}_{5}$, Fe${\;}_{1.77}$Ge and Fe${\;}_{3}$Ge impurity phases. The X-ray diffraction pattern and Rietveld refinement are shown in Figure B2. The estimated phase purity for LiFe${\;}_{6}$Ge${\;}_{4}$ was approximately 90 wt% from Rietveld refinement whilst the estimated phase fractions of LiFe${\;}_{6}$Ge${\;}_{5}$, Fe${\;}_{1.77}$Ge and Fe${\;}_{3}$Ge were 7.5, 2 and 0.5 wt%, respectively. The material adopts the *R*$\bar{3}$ *mH* space group with refined unit cell parameters of *a* = 5.0521(1) and *c* = 19.6755(4) Å which is in agreement with later works [39] rather than the *C*12*/m*1 space group identified in earlier experiments and used in the calculations for the high-throughput search. The minor phase LiFe${\;}_{6}$Ge${\;}_{5}$ is crystallographically closely related to LiFe${\;}_{6}$Ge${\;}_{4}$, the unit cell length of LiFe${\;}_{6}$Ge${\;}_{5}$ is doubled in the *c* direction relative to the LiFe${\;}_{6}$Ge${\;}_{4}$ compound. The presence of the impurity phases is potentially due to inhomogeneity or Li sublimation during welding of the Ta reaction vessels. This was minimized through the use of a large excess of Li as previously reported in the literature and care during heating [40]. X-ray diffraction patterns were also recorded over several days to confirm the stability of the compounds.

**Figure B3.**
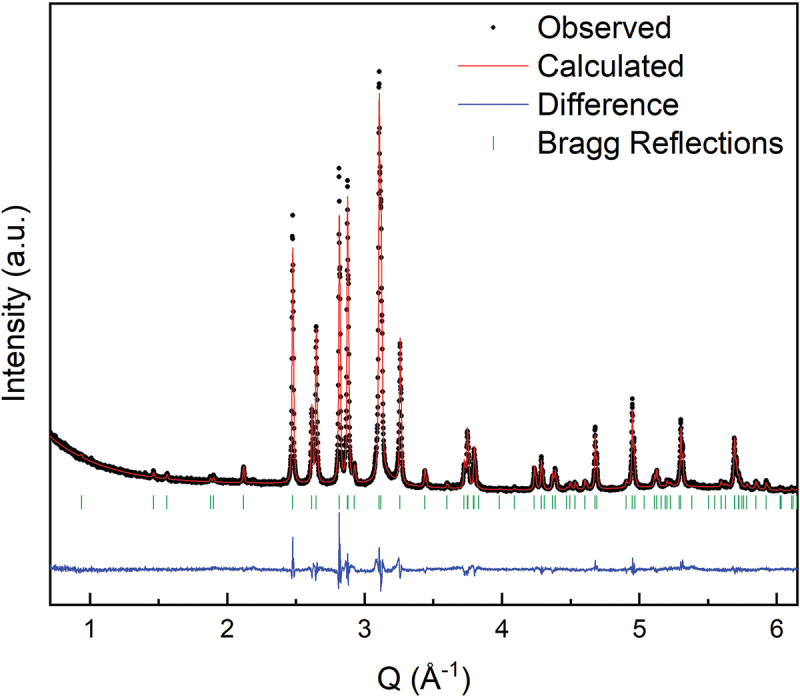
(Color online) Powder xray diffraction pattern (*λ* = 1.54 Å) and Rietveld refinement of ZrFe_6_Ge_4_. *rwp* 6.86 and *χ*^2^ 3.11. The measured diffraction data is shown in black,the calculated diffraction curve based on the structural model in red and the difference between the calculated and measured data in blue. The tick marks indicate the expected peak positions for ZrFe_6_Ge_4_.

The powder X-ray diffraction pattern of the ZrFe${\;}_{6}$Ge${\;}_{4}$ compound confirms that the material is isostructural to the LiFe${\;}_{6}$Ge${\;}_{4}$ compound with no evidence of a secondary phase. The results of the Rietveld refinement are shown in Figure B3 and the refinement gave unit cell parameters of *a* = 5.0763(1) and *c* = 20.0862(3) Å The data are in agreement with literature data previously reported for ZrFe${\;}_{6}$Ge${\;}_{4}$ [41]. Noticeable preferred orientation was observed in both compositions which was incorporated into the refinement and can be ascribed to the relatively long *c* unit cell parameter. Full details of the Rietveld refinements can be found in the tables in Appendix A.

The isothermal magnetization of the LiFe${\;}_{6}$Ge${\;}_{4}$ and ZrFe${\;}_{6}$Ge${\;}_{4}$ samples was measured at T=10 K; the results are presented in Appendix B, Figure B11a. Moreover, the temperature dependent magnetization in a magnetic field of $\mu_{0}H=0.5$ T was measured between 300 and 1100 K to investigate the influence of impurity phases such as Fe${\;}_{1.77}$Ge, Fe${\;}_{3}$Ge and Fe. The results are presented in Appendix B, Figure B11b. The results give evidence of a weak ferromagnetic or ferrimagnetic behaviour with a magnetic ordering temperature above room temperature. However, the saturated magnetization of both samples is disappointingly small, less than $1\mu_{B}$ per formula unit in a magnetic field of 5 T, a number that is considerably smaller than the value predicted by the theory. Both samples undergo significant decomposition during the measurement due to the high temperatures. However, no contribution from Fe was observed in either case (the details can be found in Appendix B).

### 4.3. Origin of the discrepancy

**Figure B4.**
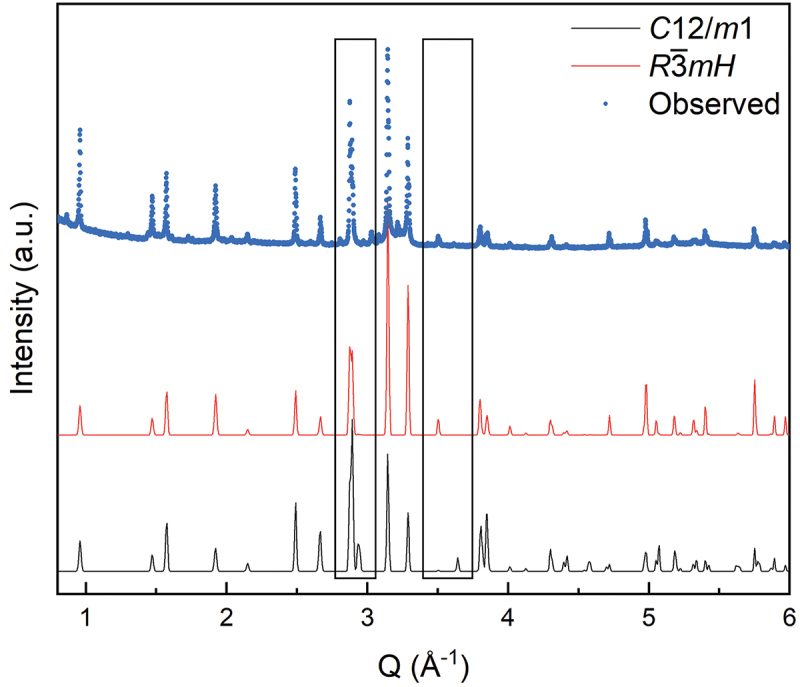
(Color online) Comparison of the simulated X-ray diffraction patterns of LiFe6Ge4 generated using the *R*3*mH* and *C*12*/m*1 space groups with the observed data for the LiFe6Ge4 sample. The black boxes highlight regions of interest in the diffraction pattern.

Since the magnetization of the synthesized Li-based compound was much smaller than theoretically predicted [16], it was necessary to investigate the origin of the discrepancy. The cause was found to be in structural effects. The space group of LiFe${\;}_{6}$Ge${\;}_{4}$, as obtained from powder X-ray diffraction, is *R*$\bar{3}$ *mH* (space group (SG) 166) [39], in contrast to the *C*12*/m*1 (space group 12) [40] (Figure B1) used in the calculations [16]. The two crystal structures are very closely related and their diffraction patterns are almost identical, as shown in Figure B4. The discrepancy between the two reported structures is likely the result of missing trigonal symmetry elements used in identifying the monocolinic *C*12*/m*1 structural model, a problem previously reported in the literature for a range of monoclinic materials [39].

LiFe${\;}_{6}$Ge${\;}_{4}$ has been reported to form both in SG 12 and SG 166; both are listed in ICSD. However, in our data-mining search [16], the latter structure was discarded due to its weak magnetic performance. To analyze the magnetism of LiFe${\;}_{6}$Ge${\;}_{4}$ (SG 166 vs SG 12) and explore the feasibility of further attempts to synthesize the material (SG 12), we calculated the total energies of the magnetic states, particularly the FM and AFM states in SG 166 and the FM state of SG 12. We found the energies of the two magnetic phases in SG 166 to be very close to each other, with the preferred AFM orientation of the magnetic moments. The lowest energy of all the considered antiferromagnetic configurations lies only 4 meV/atom below the FM phase.

When comparing the formation enthalpy of the two structures (SG 166 vs SG 12), the trigonal phase (SG 166) was found to be more stable, with a large energy difference of 252 meV/atom. This number discourages further attempts to produce the material with SC 12, proving that the *ICSD 100,061* entry is likely the result of a characterization error.

### 4.4. Calculations: changing the magnetic state

**Figure B5.**
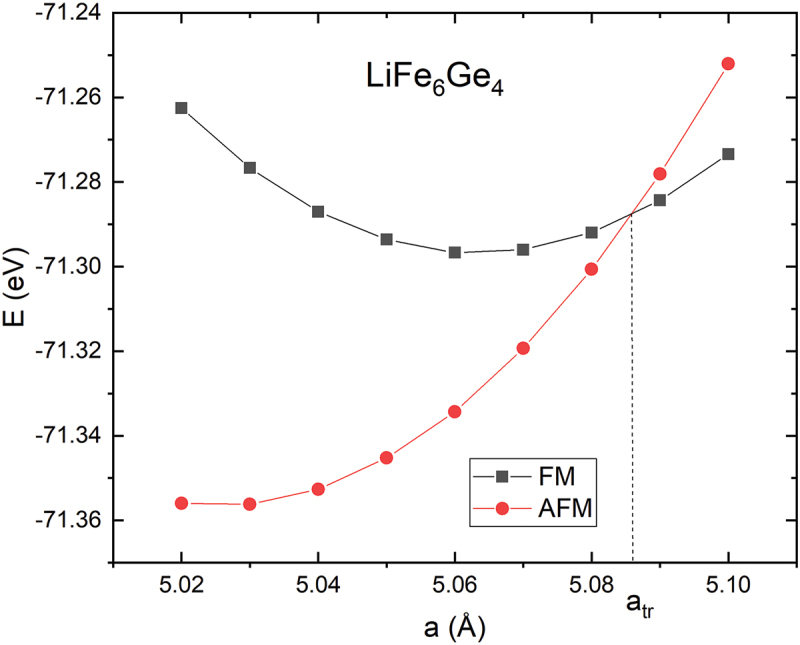
(Color online) the total energies of the FM and AFM s tates of LiFe_6_Ge_4_ − 66 at different values of *a* with a constant experimental volume of V = 145.18^°^A^3.^

As the FM state of LiFe${\;}_{6}$Ge${\;}_{4}$ in SG 12 is quite promising from the magnetic point of view, we attempted to find a way to stabilize the ferromagnetic ground state of LiFe${\;}_{6}$Ge${\;}_{4}$ in SG 166. Keeping in mind the ferromagnetic ground state of the monoclinic phase, changing the crystal structure was explored by varying the c/a-ratio at constant volume. The atomic positions in the unit cell were relaxed for a range of c/a-ratios with the experimental u.c. volume fixed (V = 145.18 $A^{3}$). It can be seen in Figure B5 that increasing the a lattice parameter by just 1–2 % makes the FM state more stable than the AFM one. At the lattice constant value where this magnetic transition occurs, $a_{\mathrm{tr}}$, the system has promising magnetization of 0.94 T and the MAE of 1.44 MJ/m${\;}^{3}$, which constitutes promising magnetic material for permanent magnet applications.

**Figure B6.**
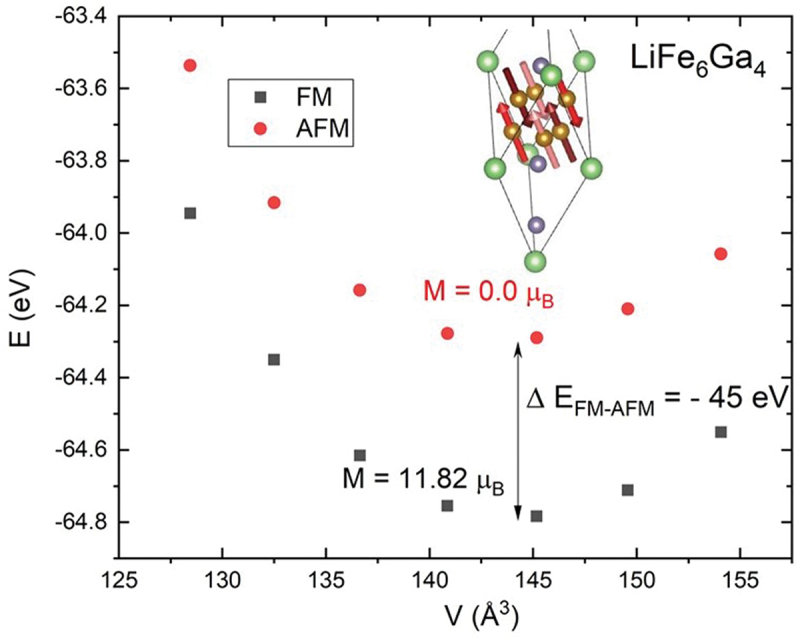
(Color online) the total energies of the FM and AFM states of LiFe_6_Ga_4_ in SG 166 at different volumes along with the magnetic moments at minimum volume and the difference in energy between the AFM and FM minima.

Since the magnetic state of LiFe${\;}_{6}$Ge${\;}_{4}$ is very sensitive to the change in the c/a-ratio and since this change can be achieved by element substitution, we considered replacing Ge fully with the neighbouring elements in the Periodic Table (with the exception of As), to pursue futther theoretical calculations. Relaxed crystal structure parameters, along with the experiential data from the current work and other available sources, are listed in Table B1. All structures were relaxed and tested for stability by calculating the formation enthalpy with respect to their elemental components (for details see the *Computational methods* section). A more sophisticated analysis which takes into account binary phases was beyond the scope of the underlying high-throughput study. LiFe${\;}_{6}$X${\;}_{4}$ with X={Si, P, Sb} (space group 166) are found to be AFM, while for X ={Al, Ga, In, Sn} the preferred ground state is FM. As an example, we show the difference in energy between the lowest in energy AFM (several AFM configurations were tested) and FM states of LiFe${\;}_{6}$Ga${\;}_{4}$ with the change in volume in Figure B6. For each of the four materials, formation enthalpy, magnetic moment, and MAE were also calculated and are listed in Table B2. The Curie temperatures are given only for the systems with large uniaxial MAE and a non-collinear state is noted with NC.

**Figure B7.**
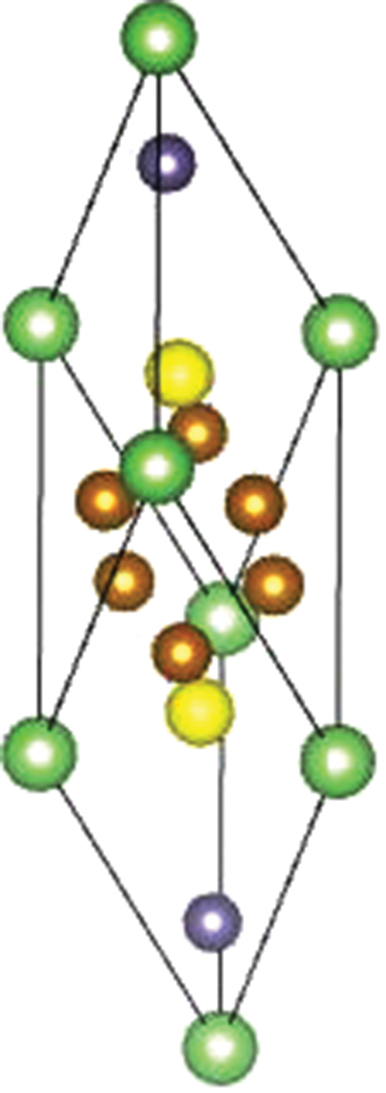
(Color online) the relaxed unit cell of LiFe_6_Al_2_Ge_2_, space group 166. The positions of Al (yellow atoms) are chosen to minimise the energy. Iron atoms are shown with the brown spheres, Ge atoms are small violet spheres, Li is denoted by the large green spheres.

LiFe${\;}_{6}$Al${\;}_{4}$ and LiFe${\;}_{6}$Ga${\;}_{4}$ are the most promising materials magnetically, as LiFe${\;}_{6}$Sn${\;}_{4}$ is quite low in magnetocrystalline anisotropy while LiFe${\;}_{6}$In${\;}_{4}$ has negative MAE (and is unstable). Partial substitution was also attempted, to investigate if lower percentages were sufficient to stabilize the ferromagnetic state. LiFe${\;}_{6}$(Ge${\;}_{0.5}$Al${\;}_{0.5}$)${\;}_{4}$ and LiFe${\;}_{6}$(Ge${\;}_{0.5}$Ga${\;}_{0.5}$)${\;}_{4}$ unit cells were constructed by substituting two of the Ge atoms with Al and Ga and choosing the positions lowest in energy out of all possible combinations, see Figure B7. The structures were relaxed and their magnetic properties are listed in Table B2. At 50% substitution of Al and Ga, the materials are ferromagnetic in their ground state, showing high magnetization and uniaxial magnetocrystalline anisotropy. Unfortunately, when performing finite temperature simulations with the ASD package, LiFe${\;}_{6}$(Ge${\;}_{0.5}$Al${\;}_{0.5}$)${\;}_{4}$ appears to be non-collinear. Similar calculations were performed for LiFe${\;}_{6}$Ge${\;}_{3}$Ga. As can be seen from Table B2, substituting 25% of Ge with Ga is not sufficient. Even though the ground state of the material is FM, it is non-collinear at higher temperatures.

Synthesis of the LiFe${\;}_{6}$(Ge${\;}_{0.5}$Ga${\;}_{0.5}$)${\;}_{4}$ and LiFe${\;}_{6}$Ga${\;}_{4}$ compounds highlighted was also attempted to validate these findings using the same synthesis method described above for the LiFe${\;}_{6}$Ge${\;}_{4}$, however, the syntheses did not yield the desired phase and instead formed a mixture of LiGa, Fe${\;}_{3}$Ga; Fe${\;}_{3}$Ge and other phases. However, given the comparable calculated values of formation enthalpy for the Ga-containing compounds, alternative synthesis methods should be explored as part of a follow up study [44].

Trying to stabilize the FM ground state and to decrease the amount of Li in the compound, Li substitution with Zr was tested. Replacing Li fully makes the ground state FM and the MAE increases considerably. However, the atomistic spin-dynamics calculations show ZrFe${\;}_{6}$Ge${\;}_{4}$ to be non-collinear at finite temperatures, see Table B2. ZrFe${\;}_{6}$Ga${\;}_{4}$ has a low $T_{C}$ = 15 K and loses the high value of the MAE.

Replacing Fe with its neighbouring 3 *d* magnetic elements was also attempted in the theoretical calculations to stabilize the FM ground state. The unit cells of Li *X*${\;}_{6}$Ge${\;}_{4}$ (*X* = Mn, Co) were relaxed both in volume and a/c-ratio. Unfortunately, both materials preserve the AFM ground state. On the other hand, replacing just one Fe with Cr or Mn makes the material FM, although the $\Delta E_{\mathrm{FM}-\mathrm{AFM}}$ values of −20 meV/atom and −10 meV/atom are not large enough to yield the desired high Curie temperature.

In Ref. [43] another material of the same family, ScFe${\;}_{6}$Ge${\;}_{4}$ (SG 166), is reported. The authors synthesised the compound and performed magnetic measurements along with computational simulations of the ground state. They also suggested MgFe${\;}_{6}$Ge${\;}_{4}$ to be a promising material, though investigated computationally only. To complement our work, we performed DFT calculations for the ground state of these two materials and the ASD simulations for the higher temperatures. The results show that even though both materials have high magnetization and MAE at 0 K, they show non-collinear spin structures when ASD calculations at elevated temperatures are performed.

As approximately 7% of the here synthesized Li-sample is LiFe${\;}_{6}$Ge${\;}_{5}$, we performed additional magnetic calculations for this compound. Similar to LiFe${\;}_{6}$Ge${\;}_{4}$, ICSD suggests two possible crystal structures for LiFe${\;}_{6}$Ge${\;}_{5}$, namely, *R*$\bar{3}$ *mH* (SG 166) and *C*12*/m*1 (SG 12). Unlike LiFe${\;}_{6}$Ge${\;}_{4}$, however, the ground states of the two structures appear to be extremely close in energy, with *C*12*/m*1 higher by 0.05 meV/u.c. only. The magnetic properties of both phases are almost the same: $M_{S}^{166}$ = 0.742 T with MAE${\;}^{166}$ = 0.89 MJ/m${\;}^{3}$ and $M_{S}^{12}$ = 0.744 T with MAE${\;}^{12}$ = 0.93 MJ/m${\;}^{3}$. As the unit cell here is doubled making it cumbersome to calculate the $J_{\mathrm{ij}}$s, we use the mean-field approximation for the $T_{C}$ calculation, as implemented in the SPRKKR code (see the *Methods* section). For LiFe${\;}_{6}$Ge${\;}_{5}$ in SG 12, a Curie temperature of 1104.7 K is obtained. The nearest-neighbour exchange interactions are strongly FM (e.g. 38 meV around 0.29 *a* for one type of Fe atoms), although there are also AFM exchange couplings further away (−8.5 meV at 0.46 *a*). Similar AFM couplings cause ZrFe${\;}_{6}$Ge${\;}_{4}$ to be NC, as we show further in the *Discussion*. Indeed, after performing the ASD simulations, we can see the non-collinear structure with a small non-zero net magnetic moment (up to 0.22 $\mu_{B}$/at.) in LiFe${\;}_{6}$Ge${\;}_{5}$ at higher temperatures.

## 5. Discussion

LiFe${\;}_{6}$Ge${\;}_{4}$ was identified as the promising candidate for PM applications in the high-throughput search. A sample was synthesized to confirm these findings with magnetic measurements. The diffraction data revealed that the compound was isostructural, adopting the *R*$\bar{3}$ *mH* space group rather than the monoclinic *C*12*/m*1 space group reported in the ICSD. Further analysis showed that the crystal structure reported in the ICSD as LiFe${\;}_{6}$Ge${\;}_{4}$ with space group 12 may be the result of missed symmetry elements during earlier analysis. Our calculations show, that SG 166 is by a large margin energetically favorable. LiFe${\;}_{6}$Ge${\;}_{4}$ (SG 166), unlike LiFe${\;}_{6}$Ge${\;}_{4}$ (SG 12), exhibits an AFM ground state. The magnetic ground state, however, can be changed by applying external pressure or by element substitution. We investigated the effect of element replacement on LiFe${\;}_{6}$Ge${\;}_{4}$‘s magnetic properties, substituting Ge with its neighboring elements, Fe with Co and Mn, and Li with Zr, Sc, and Mg. All the crystal structures were relaxed and their formation enthalpies were calculated. Where the experimental data is available, our relaxed crystal structure parameters are in good agreement with the experimental data, see Table B1.

**Figure B8.**
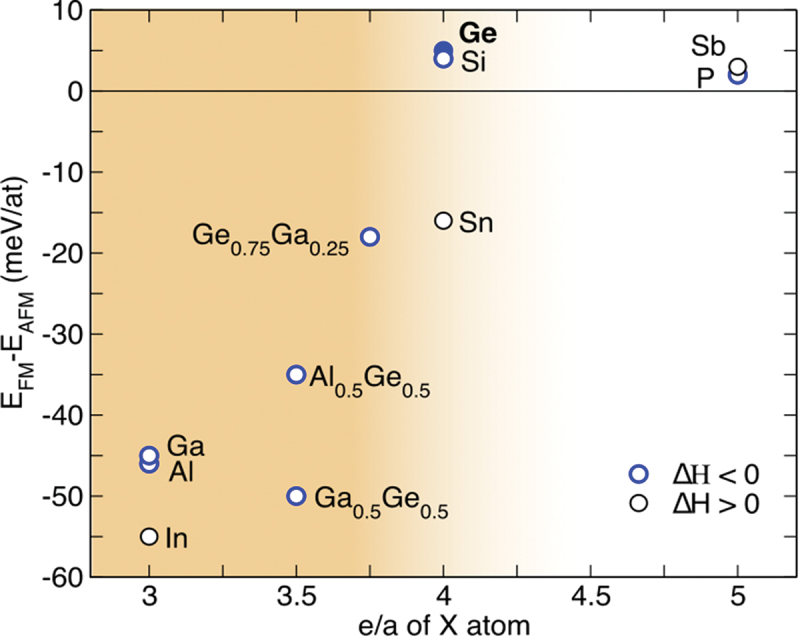
(Color online) energy difference for different dopants *X* in LiFe_6_*X*_4_ depending on the number of valence electrons (*e/a*) in the dopant *X*. Thick blue (thin black) circles denote systems with negative (positive) formation enthalpy.

In case of LiFe${\;}_{6}$ *X*${\;}_{4}$, the dopants with fewer valence electrons per atom (*e/a*) than Ge were found to favor FM ground states, see Figure B8. Replacing, for example, half of Ge by Ga lowers the energy of the FM state relative to the AFM state by around 50 meV/atom. Adding electrons, by doping with P or Sb, has the opposite effect, i.e. the AFM or at least non-FM structures are more likely. The DFT calculations were restricted to collinear spin configurations and the subsequent finite temperature ASD calculations revealed an even more complex picture, see the discussion below. Independent of the *e/a* of the dopant, when substituting Ge with heavier elements that have filled 4 *d* shells, i.e. In, Sn, and Sb, the material becomes unstable, see Table B2.

Since the choice of the magnetic element has a significant impact on the magnetic order, Fe substitution with Co and Mn was also tested. In both cases, the magnetic ground state remains AFM.

**Figure B9.**
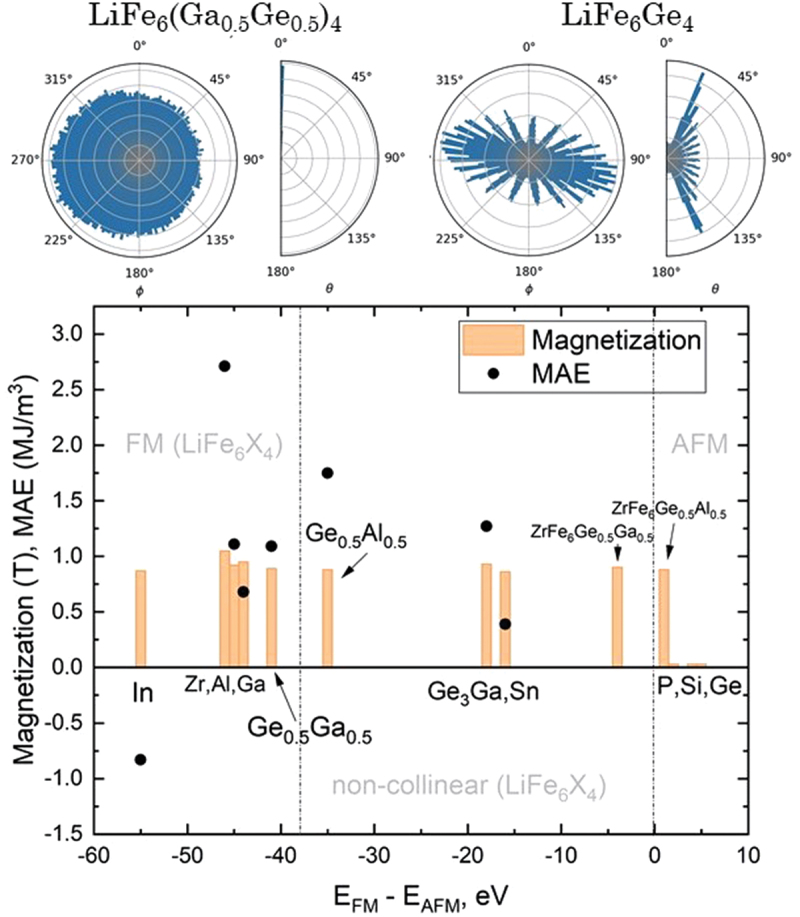
(Color online) saturation magnetization (bars) and MAE are (black dots) plotted against the difference in energy between the FM and lowest in energy AFM states for LiFe_6_*X*_4_ (*X* = Al, Si, P, Ga, Ge, in, Sn, Sb, and combinations thereof) and several Zn-based compounds. The borders between AFM, non-collinear, and FM states are shown for the Li-based materials. The AFM materials with *X* = Si, P, Ge are noted with small bars to mark their locations. Spatial distributions of the magnetic moments in the FM LiFe_6_(Ga_0.5_Ge_0.5_)_4_ and non-collinear LiFe_6_Ge_4_ at 2 K (as the result of the ASD simulations) are demonstrated in the inset above the plot. The length of the bars is proportional to the number of Fe moments with a specifi orientation and *c*-axis is directed along *θ* = 0 and *ϕ* = 0.

The saturation magnetization and the MAE of some of the materials investigated here are shown in Figure B9 as a function of the energy difference between the FM configuration and the lowest-energy AFM state. We can see that below $\Delta E_{\mathrm{FM}-\mathrm{AFM}}\approx$ −40 meV/at, LiFe${\;}_{6}$ *X*${\;}_{4}$ is a stable FM system, while above that energy difference the materials show non-collinear structures at finite temperatures (according to the ASD simulations). All the materials keep similar saturation magnetization, while the MAE varies considerably, with the highest values observed for Al and Ge. For the magnetic properties, Ge substitutions with Al or Ga seem to be the most promising options, as they keep the high magnetization and uniaxial magnetocrystalline anisotropy, and are ferromagnetic with the $T_{C}$ above 800 K.

**Figure B10.**
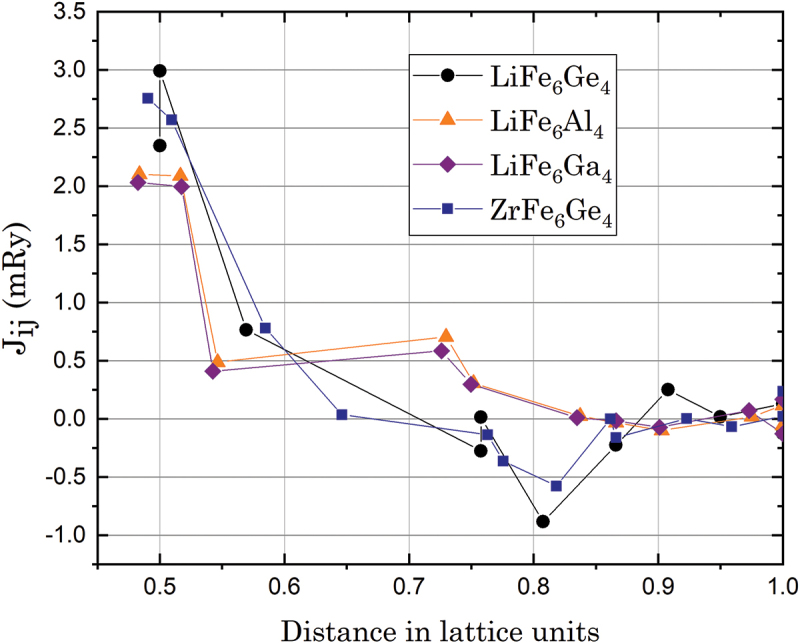
(Color online) Fe-Fe exchange parameters *J*_*ij*_ derived from first-principles calculation for the ferromagnetic LiFe_6_Al_4_ and LiFe_6_Ga_4_, non-collinear (in ASD) ZrFe_6_Ge_4_, and AFM LiFe_6_Ge_4_.

**Figure B11.**
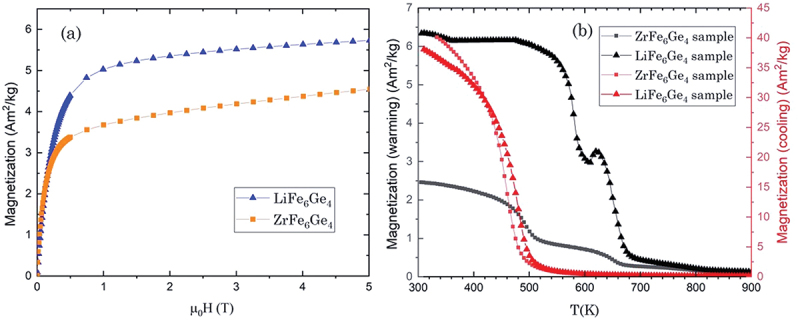
(Color online) (a) magnetization versus magnetic field for the LiFe${\;}_{6}$Ge${\;}_{4}$ and ZrFe${\;}_{6}$Ge${\;}_{4}$ samples measured at 10 K. (b) Magnetization versus temperature for the LiFe${\;}_{6}$Ge${\;}_{4}$ and ZrFe${\;}_{6}$Ge${\;}_{4}$ samples in the temperature range 300−1000 K during heating (black symbols) and cooling (red symbols) of the samples. Note that heating during the magnetometry measurements led to the decomposition of the samples; for details, Appendix B.

Exchange parameters $J_{\mathrm{ij}}$ for LiFe${\;}_{6}$ *X*${\;}_{4}$ (*X* = Al, Ga, Ge) and ZrFe${\;}_{6}$Ge${\;}_{4}$ are shown in Figure B10. The nearest-neighbour short-distance interactions (within the Fe planes) are FM for all four materials. However, the ground state is considerably affected by the long-distance (interplane) AFM terms. These AFM $J_{\mathrm{ij}}$s are present for the AFM material LiFe${\;}_{6}$Ge${\;}_{4}$ and the NC ZrFe${\;}_{6}$Ge${\;}_{4}$ system, while they are not observed for the FM LiFe${\;}_{6}$Al${\;}_{4}$ and LiFe${\;}_{6}$Ga${\;}_{4}$. Hence, for these materials, looking at the long-distance $J_{\mathrm{ij}}$s can be a useful criterion to predict their magnetic behavior at higher temperatures.

The synthesis of ScFe${\;}_{6}$Ge${\;}_{4}$ and the results from magnetic measurements in the temperature range 3–600 K were reported in Ref. [43]. The authors also presented the results of their DFT structure relaxation (using VASP) and electron structure and chemical bonding calculations (applying the augmented spherical wave method). The experimental results revealed a magnetic moment considerably smaller than the theoretical 0 K value. The authors suggest that the discrepancy may be attributed to the polycrystalline nature of the samples. We propose an alternative explanation of the data. In our higher-temperature ASD simulations, a non-collinear orientation of the spins is observed with a small non-zero total moment. The material was also recently investigated in Ref. [45] as a promising topological magnet, with the experimental data and DFT calculation pointing to an AFM ground state.

Magnetic properties of a hypothetical compound MgFe${\;}_{6}$Ge${\;}_{4}$ were also studied in Ref. [43]. The material compound was found promising due to its negative formation energy and FM ground state. However, we find that the material exhibits non-collinear behaviour when ASD simulations are performed, hence, it can not be used for PM applications [46,47].

We would like to stress that the case of LiFe${\;}_{6}$Ge${\;}_{4}$ shows the importance of an extra step being added to the high-throughput searches. For the materials reported in several crystal structures, the relative stability of those phases should be investigated, including the effect crystal structure has on the desired properties.

## 6. Conclusion

To conclude, we discovered and explained the discrepancy between the magnetic properties of LiFe${\;}_{6}$Ge${\;}_{4}$ reported in the data-mining search [16] and experimental measurements. A different crystal structure was synthesized. Further investigation suggests that the ICSD database entry overlooks the correct crystal structure and is, hence, incorrect. This outcome suggests the necessity of an extra step being added to the data-mining investigations, where the related crystal structures should analyzed.

With its AFM ground state, LiFe${\;}_{6}$Ge${\;}_{4}$ (SG 166) can not be used for permanent magnet applications. However, we find that elemental substitution can induce the promising FM ground state, thus providing the desired magnetic characteristics, with the substitution of Al or Ga for Ge providing the highest benefit.

At the same time, the LiFe${\;}_{6}$Ge${\;}_{4}$ itself can be interesting to consider for other applications, as its magnetic ground state can be easily affected by, for example, applied pressure or a slight change in its lattice parameters. This property has potential in magneto-strictive applications, including magnetocalorics.

## Appendix A. Rietveld refinement

The refined parameters for LiFe_6_Ge_4_ and Zr Fe_6_Ge_4_ are given in Tables B3 and B4 respectively.

**Table B1.** Calculated and experimental lattice parameters for LiFe${\;}_{6}$Ge${\;}_{4}$-type compounds.

Material	*a*	*c*	V	Reference
	A	A	$A^{3}$	
LiFe${\;}_{6}$Al${\;}_{4}$	5.017	19.67	429.0	current work (calc)
LiFe${\;}_{6}$Si${\;}_{4}$	4.879	18.87	389.0	current work (calc)
LiFe${\;}_{6}$P${\;}_{4}$	4.877	18.03	371.3	current work (calc)
LiFe${\;}_{6}$Ga${\;}_{4}$	5.040	19.66	432.4	current work (calc)
LiFe${\;}_{6}$Ge${\;}_{4}$	5.0521(1)	19.6755(4)	434.915(17)	current work (exp)
LiFe${\;}_{6}$Ge${\;}_{4}$	5.045	19.66	433.4	[39,40]
LiFe${\;}_{6}$(Ge${\;}_{0.5}$Al${\;}_{0.5}$)${\;}_{4}$	4.995	19.77	427.2	current work (calc)
LiFe${\;}_{6}$(Ge${\;}_{0.5}$Ga${\;}_{0.5}$)${\;}_{4}$	5.007	19.74	428.6	current work (calc)
LiFe${\;}_{6}$Ge${\;}_{3}$Ga	5.026	19.62	429.2	current work (calc)
LiFe${\;}_{6}$In${\;}_{4}$	5.379	20.95	525.0	current work (calc)
LiFe${\;}_{6}$Sn${\;}_{4}$	5.375	20.76	519.4	current work (calc)
LiFe${\;}_{6}$Sb${\;}_{4}$				current work (calc)
ZrFe${\;}_{6}$Ge${\;}_{4}$	5.061	20.19	447.9	current work (calc)
ZrFe${\;}_{6}$Ge${\;}_{4}$	5.0763(1)	20.0862(3)	448.245(14)	current work (exp)
ZrFe${\;}_{6}$Ge${\;}_{4}$	5.073	20.10	448.0	[41]
ZrFe${\;}_{6}$Ga${\;}_{4}$	5.061	20.17	447.5	current work (calc)
ScFe${\;}_{6}$Ge${\;}_{4}$	5.055	20.02	443.1	current work (calc)
ScFe${\;}_{6}$Ge${\;}_{4}$	5.066	20.01	444.8	[42]
ScFe${\;}_{6}$Ge${\;}_{4}$	5.079	20.01	447.0	[43]
MgFe${\;}_{6}$Ge${\;}_{4}$	5.065	19.86	441.3	current work (calc)

**Table B2.** Magnetic ground state, calculated saturation magnetization, MAE, Curie temperature (calculated with ASD), formation enthalpy, and the energy difference between the FM and AFM states for LiFe${\;}_{6}$ *X*${\;}_{4}$ (*X* = Al, Si, ga, Ge, Ga/Ge, in, Sn, and Sb) and ZrFe${\;}_{6}$ *Z*${\;}_{4}$ (*Z* = ga and Ge), ScFe${\;}_{6}$Ge${\;}_{4}$, and MgFe${\;}_{6}$Ge${\;}_{4}$. NC stands for the non-collinear magnetic structure.

	Ground	M${\;}_{S}$	MAE	$T_{C}$	ΔH	$\Delta E_{\mathrm{FM}-\mathrm{AFM}}$
Material	state	T	MJ/m${\;}^{3}$	K	meV/at	meV/at
LiFe${\;}_{6}$Al${\;}_{4}$	FM	0.92	1.11	850	−155.5	−46
LiFe${\;}_{6}$Si${\;}_{4}$	AFM				−361.7	+4
LiFe${\;}_{6}$P${\;}_{4}$	AFM				−373.1	+2
LiFe${\;}_{6}$Ga${\;}_{4}$	FM	0.95	0.68	885	−120.9	−45
LiFe${\;}_{6}$Ge${\;}_{4}$	AFM					+5
LiFe${\;}_{6}$(Ge${\;}_{0.5}$Al${\;}_{0.5}$)${\;}_{4}$	FM	0.88	1.75	NC	−205.7	−35
LiFe${\;}_{6}$(Ge${\;}_{0.5}$Ga${\;}_{0.5}$)${\;}_{4}$	FM	0.89	1.09	820	−157.6	−50
LiFe${\;}_{6}$Ge${\;}_{3}$Ga	FM	0.93	1.27	NC	−197.5	−18
LiFe${\;}_{6}$In${\;}_{4}$	FM	0.87	−0.83	730	98	−55
LiFe${\;}_{6}$Sn${\;}_{4}$	FM	0.86	0.39	NC	51.4	−16
LiFe${\;}_{6}$Sb${\;}_{4}$	AFM				47.5	+3
ZrFe${\;}_{6}$Ge${\;}_{4}$	FM	1.05	2.71	NC	−92.4	−44
ZrFe${\;}_{6}$Ga${\;}_{4}$	FM	0.99	0.79	15		−37
ScFe${\;}_{6}$Ge${\;}_{4}$	FM	1.00	1.87	NC	−263.9	−67
MgFe${\;}_{6}$Ge${\;}_{4}$	FM	0.93	1.87	NC	−110.1	−70

**Table B3.** Refined parameters for LiFe${\;}_{6}$Ge${\;}_{4}$ derived from powder X-ray diffraction data and Rietveld refinement.

Atom	Wyckoff Position	*x*	*y*	*z*	Occupancy	beq
Li	3 *a*	0	0	0	1	1
Fe	18 *h*	0.4992(3)	−0.4992(3)	0.1047(1)	1	3.971(75)
Ge1	6 *c*	0	0	0.1259(2)	1	2.931(91)
Ge2	6 *c*	0	0	0.3316(2)	1	2.931(91)

**Table B4.** Refined parameters for ZrFe${\;}_{6}$Ge${\;}_{4}$ derived from powder X-ray diffraction data and Rietveld refinement.

Atom	Wyckoff Position	*x*	*y*	*z*	Occupancy	beq
Zr	3 *a*	0	0	0	1	0.970(57)
Fe	18 *h*	0.4965(3)	−0.4965(3)	0.1025(1)	1	1.754(42)
Ge1	6 *c*	0	0	0.1318(9)	1	0.904(35)
Ge2	6 *c*	0	0	0.3328(9)	1	0.904(35)

## Appendix B. Experiment: magnetism

Isothermal magnetization curves at 10 K of the LiFe${\;}_{6}$Ge${\;}_{4}$ and ZrFe${\;}_{6}$Ge${\;}_{4}$ samples are shown in Figure B11. The results give evidence of a weak ferromagnetic or ferrimagnetic behaviour. However, the saturated magnetic moment of both samples is disappointingly small, less than 1 $\mu_{B}$ per formula unit in a magnetic field of 5 T, a number that is considerably smaller than the value predicted by the theory.

The magnetic properties of the LiFe${\;}_{6}$Ge${\;}_{4}$ and ZrFe${\;}_{6}$Ge${\;}_{4}$ samples were also measured in a magnetic field 0.5 T between 300 and 1100 K to investigate possible contributions from impurity phases such as Fe${\;}_{1.77}$Ge, Fe${\;}_{3}$Ge and Fe. Both samples undergo significant decomposition during the magnetometry measurements due to the high temperatures, which makes further analysis of the magnetic behavior challenging. The decomposition was confirmed by X-ray diffraction after magnetometry measurements and differential scanning calorimetry measurements of pristine samples following the same temperature protocol.

The temperature dependent magnetization of the ZrFe${\;}_{6}$Ge${\;}_{4}$ sample revealed three magnetic transition events upon warming, two of which correspond to the known Curie temperatures of Fe${\;}_{1.77}$Ge (490 K) and Fe${\;}_{3}$Ge (650 K). These phases emerge during the heating and were not observed in the initial X-ray diffraction measurements. A third weaker change in magnetisation was observed at around 805 K, however, it is challenging to identify the phase formed during this stage of the decomposition. No contribution from Fe was observed in the magnetometry data or in the X-ray diffraction results. Upon cooling from 1100 K, the result from the magnetometry measurement revealed only one magnetic transition at about 460 K, indicating single phase behavior and complete decomposition of the ZrFe${\;}_{6}$Ge${\;}_{4}$ and Fe${\;}_{3}$Ge phases.

Similarly, the LiFe${\;}_{6}$Ge${\;}_{4}$ sample underwent decomposition and Li loss during the high temperature magnetometry. After the magnetic measurement, X-ray diffraction revealed that the desired LiFe${\;}_{6}$Ge${\;}_{4}$ phase had entirely decomposed. However, the magnetic curve measured upon heating revealed two magnetic transitions (∼ 575K and ∼ 650K). The first transition most probably relates to an Fe${\;}_{2-x}$Ge phase [45] or a decomposition product, while the second transition corresponds to the Fe${\;}_{3}$Ge phase, also observed in the ZrFe${\;}_{6}$Ge${\;}_{4}$ sample.

Using results from the Rietveld refinement of the LiFe${\;}_{6}$Ge${\;}_{4}$ sample and published data for the saturation magnetization at 300 K of the ferromagnetic impurity phases Fe${\;}_{1.77}$Ge (∼80 Am${\;}^{2}$/kg) and Fe${\;}_{3}$Ge (∼140 Am${\;}^{2}$/kg) [45, 46], it is possible to estimate their contribution to the measured magnetic moment at room temperature. With 2 and 0.5 wt% of Fe${\;}_{1.77}$Ge and Fe${\;}_{3}$Ge, respectively the contribution of the ferromagnetic impurity phases to the measured magnetic moment at 300 K amounts to approx 35 %.
